# Desire for self-control and task performance: A plan is a key

**DOI:** 10.3389/fpsyg.2022.1011559

**Published:** 2022-10-14

**Authors:** Liad Uziel, Mindi Price, Jessica L. Alquist

**Affiliations:** ^1^Department of Psychology, Bar-Ilan University, Ramat-Gan, Israel; ^2^Department of Psychological Sciences, Texas Tech University, Lubbock, TX, United States

**Keywords:** self-control, desire for self-control, trait self-control, implementation intention, goals, performance

## Abstract

This research reports two studies testing whether implementation intentions can improve performance for people high in Desire for Self-Control (DSC). DSC reflects a wish to have more self-control and was previously found to be associated with impaired self-control performance. We hypothesized that implementation intentions could reverse the effect by providing clear guidance on how to handle self-control challenges. Two experiments (*N*s = 175, 302) tested this hypothesis using different self-control tasks and manipulated and measured DSC. Results from both studies showed that DSC interacts with implementation intentions, such that among individuals high in DSC (but not among individuals low in DSC), implementation intentions were helpful in improving self-control performance. Implications, limitations, and future directions are discussed.

## Introduction

The numerous benefits associated with high self-control make it a desirable characteristic (Tangney et al., 2004; Richmond-Rakerd et al., 2021). Research often concludes that more self-control is better (Baumeister and Alquist, 2009; Duckworth, 2011), and the popular media praise the advantages of high self-control (Tugend, 2010). Moreover, educational programs are designed to foster self-control in young children (Diamond et al., 2007), and laypeople consider self-control an underdeveloped personal strength (Park et al., 2006) and attribute their failures in life to shortages in self-control (American Psychological Association [APA], 2012).

In response to experiencing shortages in self-control, individuals often express a wish for more self-control. The *desire for self-control* (DSC) has been conceptualized as a “desire to be better able to change and consciously redirect one’s cognitions, impulses, emotions, performance, and other behaviors” (Uziel and Baumeister, 2017, p. 694). DSC reflects a sense that one does not have enough self-control to meet current goals and is considered a response to societal pressure to display higher levels of self-control.

In a recent study, Uziel et al. (2021) reported that DSC was associated with fear of failure, prevention focus, and low general self-efficacy. DSC increased in the face of demands to display self-control and it predicted the willingness to enroll in training programs designed to improve self-control (see also Ecker et al., 2021). DSC is negatively correlated with trait self-control, indicating that individuals with low self-control generally experience a stronger desire. Notwithstanding, the correlation is moderate, implying that these constructs may play different roles in predicting self-control behavior (Uziel and Baumeister, 2017; Uziel et al., 2021).

In the context of task performance, DSC was found to predict impairments in self-control performance, even after controlling for trait self-control. Across different types of self-control tasks, higher DSC (manipulated or measured) was associated with a reduction in self-control ability (Uziel and Baumeister, 2017). When faced with challenging self-control tasks, individuals with a strong DSC are inclined to perceive a gap between their actual and ought selves, which they feel they cannot overcome. This leads them to experience diminished motivation and to give up on tasks.

Notwithstanding, strong DSC does not always lead to a reduction in self-control performance. When the challenge is simple, DSC appears not to impair performance, probably because the path to success is relatively clear (Uziel and Baumeister, 2017). Relatedly, in the context of the Covid-19 pandemic, a recent study found that higher DSC predicted stronger adherence to (United States’s) Center for Disease Control guidelines, indicating that when clear instructions are provided, DSC may facilitate goal-directed behavior (Rodriguez et al., 2021).

The present investigation seeks to extend these findings and explore the conditions that assist individuals with a strong DSC in not giving up on difficult tasks. Building on previous findings, we reasoned that DSC leads to impairments in performance partly because it does not translate into specific action plans, which are needed when tackling complex problems. Thus, we sought to explore whether structuring one’s responses through implementation intentions could alleviate the difficulty by breaking it into predictable pre-planned action plans.

Implementation intentions are ‘if-then plans’ specifying “when, where, and how the person will instigate responses that promote goal realization” (Gollwitzer and Sheeran, 2006, p. 70). By forming implementation intentions, individuals automatize their responses in the face of hardships, reduce the self-regulatory burden associated with goal attainment, and thwart potential distractors (see Gollwitzer, 1999, for a review). Implementation intentions were suggested as a bridge between intentions and goal attainment following evidence that simply setting goals does not consistently translate into successful implementations. A meta-analysis has documented that implementation intentions have a medium-to-large effect on promoting successful goal completion and that they are especially effective in shielding goal striving from unwanted influences (Gollwitzer and Sheeran, 2006). Moreover, past research has documented that they are especially effective for ‘difficult-to accomplish’ goals (Gollwitzer and Brandstätter, 1997).

We report two studies where we had participants work on relatively difficult self-control tasks. In Study 1, participants’ level of DSC was experimentally manipulated, whereas in Study 2 DSC was measured. In both studies, participants were randomly assigned to either an implementation intention condition or a control condition before performing a task. We expected that implementation intentions would assist individuals with high DSC in overcoming their difficulties and performing better on the tasks (even after controlling for trait self-control).

Materials, data, and code of both studies are available at the Open Science Framework (OSF)^1^.

## Study 1

In Study 1, participants were experimentally induced to experience high (vs. low) DSC before facing a relatively difficult self-control task. Before starting the task, half of the participants were instructed to follow an implementation intention plan (vs. a no-plan control group). We expected an interaction between DSC and implementation intention, such that the effect of implementation intentions would be more beneficial among individuals high in DSC (compared with their effect among individuals low in DSC). *A-priori* power analysis advised that a sample of *N* = 152 is needed to detect with 80% power a small-medium effect, η_p_^2^ = 0.05 (G*Power; Faul et al., 2009). Our final sample exceeded this.

### Materials and methods

#### Participants

Participants were recruited through Prolific online platform for a study on personality and language processing. The initial sample included 180 participants, but 5 were removed (after failing attention checks or providing missing or non-sense responses on the DSC manipulation) setting the final sample at *N* = 175 (118 Female, 56 Male, 1 Non-binary; *M*_age_ = 41.42, *SD* = 13.73; 173 British).

#### Instruments

##### Trait self-control

The brief (13-item) version of the Trait Self-Control Scale (Tangney et al., 2004; e.g., “I am good at resisting temptation”; α = 0.88) was applied.

##### Desire for self-control manipulation

DSC was manipulated using a procedure detailed in Uziel and Baumeister (2017). In the high DSC condition, participants are first asked to explain why high self-control is positive, important, and beneficial. Following, they are asked to detail one or two incidents in their lives where they needed high self-control but failed to do so. In the low DSC, participants follow the same two phases, focusing on the limitations of high self-control and describing situations where they needed less self-control but failed to do so. As a manipulation check, participants reported their current level of DSC using the DSC scale (Uziel and Baumeister, 2017; α = 0.88; See Study 2 for details on this scale).

##### Implementation intention manipulation

Implementation intentions specify when, where and how goal striving is advanced. They follow this basic design: “If situation Y is encountered, then I will initiate goal-directed behavior X!” (Gollwitzer and Sheeran, 2006). In the present study, before starting the task, participants in the implementation intention condition were asked to read, memorize, and repeat aloud the following sentence: “If finding a name of a month in the matrix is difficult, then I will try again.” Participants in the control condition continued with no specific guidance.

##### Word search task

Self-control ability was measured through performance on a letter grid. Participants were told that the names of months are embedded in a 23-letter by 16-letter grid. Month names could appear in any direction (including backward). Participants were told that they can work on the task until they feel they found all the embedded words. The number of words found was our main dependent measure. Performing well on this task requires persistence and an ability to shift frames of mind. To further the role of self-control in performance, participants were told that there are between 1 and 12 names of months, thus creating a temptation to quit before finding all names while saving face. In practice, there were 10 names. We used the two non-existing names of months as an indication of participants’ integrity in their reports (participants had to write down the names found, not mark them on the grid). After completing the task, participants rated how difficult and how effortful the task has been for them on a 1-*not at all* to 7-*very much* scale.

#### Procedure

After signing a consent form, participants completed a *trait self-control* scale. Next, they were randomly assigned to a *high* or *low DSC condition*. They were then introduced to our *measure of self-control—*a word search task, whereby 10 names of months were embedded in a letter matrix. After learning about the task, but before working on it, participants were assigned to an *implementation intention condition* (vs. a control condition), whereby they expressed an intention to try again if they find the task difficult. Participants then proceeded to work on the task. After a brief demographic questionnaire, they were debriefed and thanked.

#### Data analysis

Data analysis (descriptive statistics, *t*-tests, ANCOVA) was completed using SPSS 27.

### Results and discussion

#### Manipulation and attention checks

##### Desire for self-control

We verified that the DSC manipulation was effective by comparing the mean scores on the DSC scale between the high desire condition (*M* = 3.97, *SD* = 0.69) and the low desire condition (*M* = 3.69, SD = 0.73), *t*(173) = 2.63, *p* = 0.009, *d* = 0.397, 95% CI [0.097, 0.696].

##### Task difficulty

Mean rating of task difficulty was compared to the scale’s neutral mid-point (of 4). Results indicated that, as planned, participants perceived the task as relatively difficult, *M* = 5.17, *SD* = 1.43, *t*(174) = 10.78, *p* < 0.001, *d* = 0.815, 95% CI [0.643, 0.985], and as relatively effortful, *M* = 5.43, *SD* = 1.42, *t*(174) = 13.40, *p* < 0.001, *d* = 1.013, 95% CI [0.829, 1.189].

##### Implementation intention

To verify that participants were attentive to the implementation intention instructions, we asked participants in the implementation intention condition whether they read the instructions. All participants approved.

#### Main analysis

**Figure 1:**
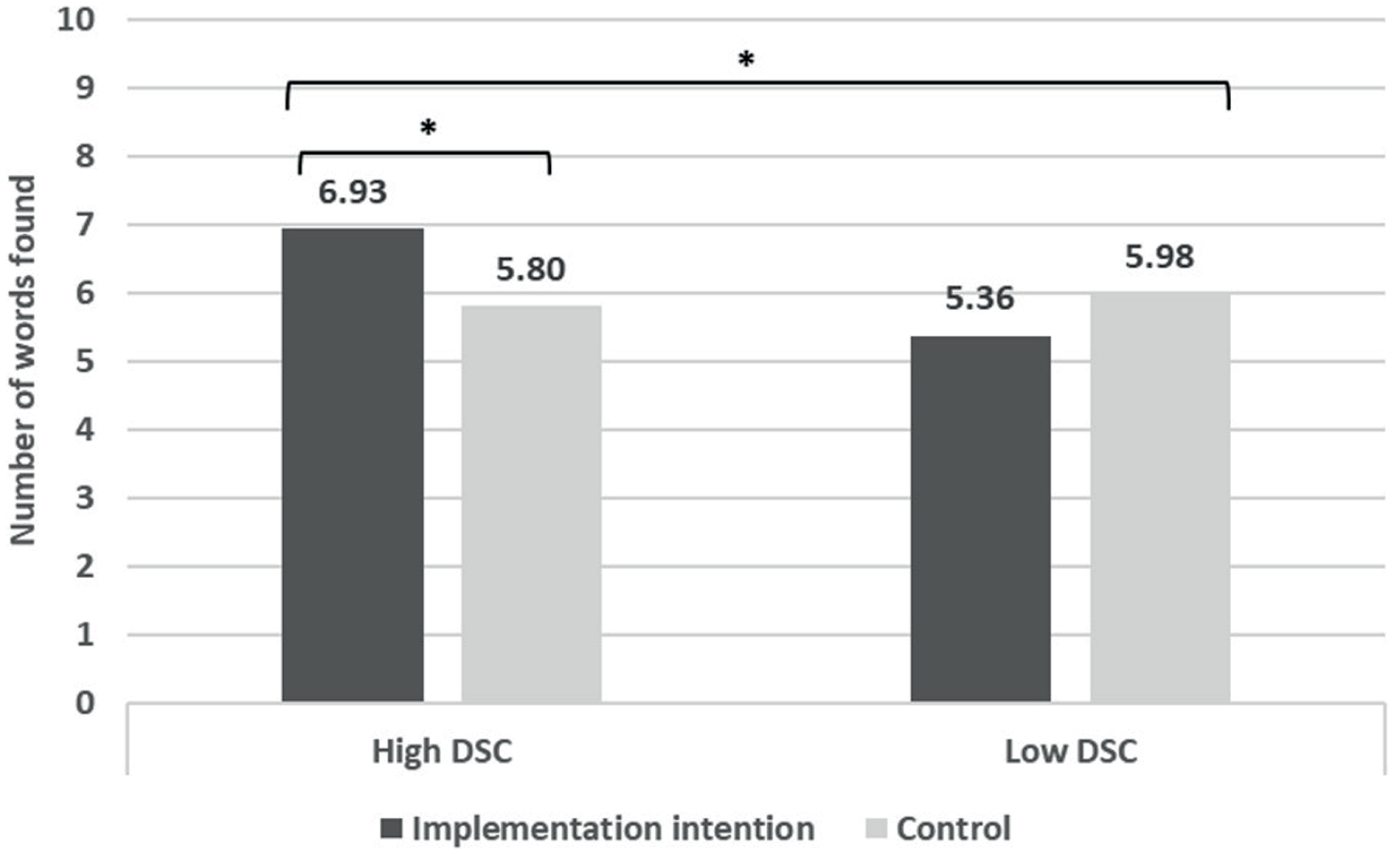
Performance on a self-control task as a function of desire for self-control and implementation intention (Study 1). DSC, Desire for self-control. * = *p* < 0.05.

Participants correctly identified *M* = 6.01 (*SD* = 2.47) of the 10 names of months embedded in the grid. Our main analyses explored how DSC and implementation intention affected performance on this task. As presented in Table 1, an ANCOVA (controlling for trait self-control^2^) yielded (only) a significant interaction, *F*(1,170) = 5.90, *p* = 0.016, η_p_^2^ = 0.034, 90% CI [0.003, 0.088]. Probing the interaction (Figure 1) revealed that for individuals in the high DSC condition, implementation intention (vs. control) improved task performance, *M* = 6.93, *SD* = 2.25 (vs. *M* = 5.80, *SD* = 2.76), *t*(83) = 2.06, *p* = 0.042, *d* = 0.448, 95% CI [0.016, 0.877]. Individuals low in DSC performed slightly worse in the implementation intention condition than in the control condition, but the effect was not significant, *M* = 5.36, *SD* = 2.51 (vs. *M* = 5.98, *SD* = 2.15), *t*(88) = 1.27, *p* = 0.208, *d* = 0.268, 95% CI [−0.149, 0.683].

**TABLE 1 Study 1:** ANCOVA for predicting performance on a self-control task by desire for self-control and implementation intention (controlling for trait self-control).

Predictor	Sum of Squares	*df*	Mean square	*F*	*p*	η*_p_*^2^	90% *CI*
TSC	3.51	1	3.51	0.595	0.441	0.003	0.000, 0.032
DSC^a^	20.06	1	20.06	3.407	0.067	0.020	0.000, 0.066
II^b^	2.74	1	2.74	0.466	0.496	0.003	0.000, 0.030
DSC*II	34.73	1	34.73	5.897	0.016	0.034	0.003, 0.088
Error	1001.06	170	5.89				

*N* = 175.

TSC, Trait self-control; DSC, Desire for self-control; II, Implementation intention.

a 0, Low desire for self-control; 1 = High desire for self-control.

b 0, No implementation intention; 1, Implementation intention.

Study 1 showed that individuals experiencing high DSC benefit from making implementation intentions before working on demanding self-control tasks. The predictability and advanced planning before facing hardship assisted them in translating their desire into productive self-controlled behavior. The findings also imply that for some individuals (individuals low in DSC) implementation intentions may not produce desirable effects.

## Study 2

Study 2 sought to extend and generalize the findings. To this end, we had a larger sample and a new self-control task. In addition, we were focused on individuals’ chronic levels of desire for self-control. Although DSC can increase momentarily, it is a higher hurdle when experienced chronically, thus we were interested in the experience of individuals who typically possess high DSC. Like in Study 1, we controlled for participants’ trait self-control to isolate the effect of *desire* for self-control (*cf.* Uziel and Baumeister, 2017). Another control was general self-efficacy, reflecting individuals’ overall belief about their competence across a wide variety of situations and tasks (e.g., Chen et al., 2001). Past findings have associated self-efficacy with self-control performance (e.g., Tangney et al., 2004; Chow et al., 2015; Graham et al., 2017; Liu et al., 2020). Therefore, akin to related studies (e.g., de Ridder et al., 2020), and given our goal of understanding the unique role of our focal variables (DSC and implementation intentions), we sought to monitor and control for variance stemming from such a general sense of being efficacious.

### Materials and methods

#### Participants

Participants were recruited through Mturk online platform for a study on personality and problem-solving. Given our wish to increase the power from Study 1 to detect smaller effects and considering a power analysis that advised that 309 participants are required to detect with 80% power a small effect, η_p_^2^ = 0.025 (G*Power; Faul et al., 2009), the initial sample included 320 participants. Having removed 18 participants after failing attention checks or writing clear non-sense responses on the anagrams task, the final sample was set at *N* = 302 (146 Female, 154 Male, 2 Non-binary; *M*_age_ = 37.73, *SD* = 11.18; all American).

#### Instruments

##### Desire for self-control

Participants completed the 8-item DSC scale (Uziel and Baumeister, 2017). The scale asks about motivation to have more control over impulses, emotions, cognitions, and behaviors (e.g., “I want to be better able to resist temptations”). In its trait version, participants describe themselves “in general” using a 5-point scale (1-*strongly disagree* to 5-*strongly agree*). Reliability was good (α = 0.91).

##### Trait self-control

Like in Study 1, we used the Trait Self-Control Scale (Tangney et al., 2004; α = 0.89).

##### General self-efficacy

The General Self-Efficacy Scale (Chen et al., 2001) measures one’s perception of how well one can perform across a variety of situations. The scale consists of 8 items (e.g., “Even when things are tough, I can perform quite well”), and responses are on a 5-point Likert-type scale (from 1-*strongly disagree* to 5-*strongly agree*; α = 0.92).

##### Implementation intention manipulation

Implementation intention was manipulated using a similar procedure to that of Study 1. Participants had to read, memorize, and repeat aloud the sentence: “If solving one anagram is difficult, then I will try again.” Participants in the control condition continued with no specific guidance.

##### Anagrams task

Anagrams are a typical self-control measure (e.g., Hagger et al., 2010), requiring persistence in the face of hardship. There were 40 5-letter anagrams and participants were instructed to solve as many as they can after learning that some of the anagrams may be unsolvable (in practice, all were solvable). Telling participants there could be unsolvable anagrams was meant to increase the temptation to quit while saving face. Our dependent variable was the number of anagrams solved correctly. After completing the task, participants rated how difficult and how effortful the task has been for them on a 1-*not at all* to 7-*very much* scale.

#### Procedure

Following a consent form, participants completed several scales: *DSC, trait self-control,* and *general self-efficacy*. Next, they were introduced to our *measure of self-control—* an anagram task, comprised of 40 (5-letter) anagrams. After learning about the task, but before working on it, participants were assigned to an *implementation intention condition* (vs. a control condition), whereby they expressed an intention to try again if they find the task difficult. Participants then proceeded to work on the task. After a brief demographic questionnaire, they were debriefed and thanked.

#### Data analysis

Data analysis (descriptive statistics, *t*-tests, regression) was completed using SPSS 27.

### Results

#### Manipulation and attention checks

##### Task difficulty

Mean rating of task difficulty was compared to the scale’s neutral mid-point (of 4). Results indicated that, as planned, participants perceived the task as relatively difficult, *M* = 4.88, *SD* = 1.63, *t*(301) = 9.38, *p* < 0.001, *d* = 0.540, 95% CI [0.419, 0.660], and as relatively effortful, *M* = 5.34, *SD* = 1.55, *t*(301) = 15.01, *p* < 0.001, *d* = 0.864, 95% CI [0.729, 0.993].

##### Implementation intention

To verify that participants were attentive to the implementation intention instructions, we asked participants in the implementation intention condition whether they read the instructions. All participants approved, except one (who was excluded from the analysis, see *Participants*).

#### Main analysis

**Figure 2:**
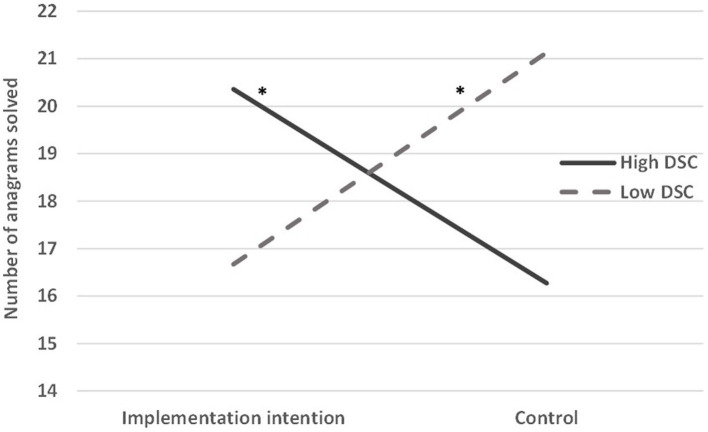
Performance on a self-control task as a function of desire for self-control and implementation intention (Study 2). DSC, Desire for self-control. * = *p* < 0.05.

Participants correctly solved *M* = 18.37 (*SD* = 12.14) of the 40 anagrams. Our main analysis explored how DSC and implementation intention affected performance on this task. We tested this with linear hierarchical regression, entering in Step 1 the predictors and the covariates (trait self-control and general self-efficacy^3^), and in Step 2 the interaction term between DSC and implementation intention. Step 1 (Δ*R*^2^ = 0.001, *p* = 0.997) revealed no main effects, *t*s < 1. Step 2 of the analysis (Δ*R*^2^ = 0.030, *p* = 0.003; presented in Table 2) revealed a negative effect for DSC, *b* = −2.676, *SEb* = 1.19, 95% CI [−5.017, −0.334], *t*(296) = −2.25, *p* = 0.025, η_p_^2^ = 0.017. Importantly, this effect was qualified by a significant interaction between DSC and condition, *b* = 4.713, *SEb* = 1.56, 95% CI [1.640, 7.785], *t*(296) = 3.02, *p* = 0.003, η_p_^2^ = 0.030. Probing the interaction (Figure 2) revealed that for individuals high in DSC (+1*SD*), implementation intention improved performance, *b* = 3.975, *SEb* = 1.98, 95% CI [0.080, 7.869], *t*(296) = 2.01, *p* = 0.046, η_p_^2^ = 0.013. Conversely, among individuals low in DSC (-1*SD*), implementation intention impaired performance, *b* = −4.547, *SEb* = 1.99, 95% CI [−8.471, −0.623], *t*(296) = −2.28, *p* = 0.023, η_p_^2^ = 0.017.

**Table 2:** Study 2: Regression analysis for predicting performance on a self-control task by desire for self-control and implementation intention (controlling for trait self-control and general self-efficacy).

Parameter	*B*	*SE*	95% *CI*	*t*	*p*
TSC	0.239	1.188	−2.10, 2.58	0.201	0.841
GSE	0.128	1.141	−2.12, 2.37	0.112	0.911
DSC	−2.676	1.190	−5.02, −0.33	−2.249	0.025
II^a^	−0.286	1.398	−3.04, 2.46	−0.205	0.838
DSC*II	4.713	1.561	1.64, 7.79	3.018	0.003

*N* = 302.

*R*^2^ = 0.030, *p* = 0.003.

TSC, Trait self-control; GSE, General self-efficacy; DSC, Desire for self-control, II, Implementation intention.

a 0, No implementation intention; 1, Implementation intention.

## General discussion

Wishing for more self-control is a natural consequence of societal pressure to conform, excel, and act in a civilized manner. However, consistently displaying high self-control is challenging, and people often fail (Baumeister et al., 2007), leading them to experience an increased desire for self-control (Uziel, 2018; Ecker et al., 2021). However, this desire is associated with fear of failure, prevention focus, and an overall sense of low efficacy (Uziel et al., 2021). Individuals wishing for more self-control often face an ironic reality whereby they are inclined to perform poorly especially when self-control is most needed. In such contexts, they adopt a debilitating state of mind making them feel inapt and more likely to quit rather than persist (Uziel and Baumeister, 2017).

The present investigation was set to explore whether this pattern can be broken. The two studies reported provide evidence that it can. Study 1 showed that participants experiencing a transient high DSC can improve their performance on a relatively difficult self-control task if they make an implementation intention guiding them to persist in the face of difficulty. In Study 2, the effect showed again, using a different task, among individuals who are chronically high in DSC. In both studies, the effects held controlling for trait self-control and (in Study 2) general self-efficacy, thereby indicating that the impact of DSC does not stem from its association with chronic abilities and beliefs, but with the desire to do better.

For individuals with a high DSC, implementation intentions appear to structure the task and make it more predictable and thus manageable. It may well be the case that the simplicity and directness of the ‘if-then’ plans (Gollwitzer and Brandstätter, 1997) what makes them an efficient mean among individuals high in DSC given their proneness to be overwhelmed by demanding situations. Breaking those into predictable contingencies makes them appear more controllable. This finding is consistent with Uziel and Baumeister’s (2017) finding that DSC does not impair performance on simple tasks and that DSC is associated with self-reported adherence to CDC guidelines during the Covid-19 pandemic (Rodriguez et al., 2021).

Somewhat unexpectedly, in Study 2 (alongside a non-significant trend in Study 1), individuals low in DSC were negatively impacted by forming implementation intentions. The literature does not often report negative effects of implementation intentions (Gollwitzer, 1999; Prestwich and Kellar, 2014), however, the present research points to the possibility that implementation intentions could impair performance under some conditions. Individuals low in DSC, by definition, are happy where they are and do not wish to further channel their behavior into structured patterns. They may resist change in a more controlled direction, either because they do not value self-control or because they consider their current level of self-control satisfactory. In both cases, having to follow such patterns reduced their level of engagement and subsequently their performance. Future studies should explore the basis for this effect, addressing their mental process while following implementation intentions.

Future studies should also address some of the limitations of the present study. First, in both studies, the tasks were word-based, and there is room to extend the finding to other self-control challenges. Relatedly, although the anagrams task is a fairly common self-control measure (e.g., Hagger et al., 2010), the word search task is a less common measure (which nonetheless builds on the same principle of persistence in the face of hardship), and thus may require further validation. Second, by their nature, the experiments involved short-term behavior in relatively controlled settings. Future studies should expand the investigation to real-life behavior, longer periods of time, and self-determined goals. Third, future studies may place more emphasis on mediating processes, especially the role of task-specific self-efficacy (*cf.* Uziel and Baumeister, 2017), in order to add insights on the mechanisms deriving the effects we observed.

Taken together, this research presented evidence that by structuring goals into simple if-then plans, individuals high in DSC may overcome their self-defeating approach to challenges.

## Data availability statement

The datasets presented in this study can be found in online repositories. The names of the repository/repositories and accession number(s) can be found at: https://osf.io/kvnqt/.

## Ethics statement

The studies involving human participants were reviewed and approved by Ethics committee at the Department of Psychology, Bar-Ilan University. The patients/participants provided their written informed consent to participate in this study.

## Author contributions

LU, MP, and JLA: idea generation, running the studies, analyzing the results, and writing the report. All authors contributed to the article and approved the submitted version

## Funding

Preparation of this manuscript was supported by a grant from the United States–Israel Binational Science Foundation (BSF), Jerusalem, Israel.

## Conflict of interest

The authors declare that the research was conducted in the absence of any commercial or financial relationships that could be construed as a potential conflict of interest.

## Publisher’s note

All claims expressed in this article are solely those of the authors and do not necessarily represent those of their affiliated organizations, or those of the publisher, the editors and the reviewers. Any product that may be evaluated in this article, or claim that may be made by its manufacturer, is not guaranteed or endorsed by the publisher.

## Footnotes

^1^ https://osf.io/kvnqt/

^2^Conclusions were not affected by removing trait self-control from the analysis.

^3^Conclusions were not affected by removing trait self-control and general self-efficacy from the analysis.
